# Alteration of Endocrine Hormones and Antibody Responses in Different Spectrum of Tuberculosis Disease

**DOI:** 10.3389/fimmu.2022.849321

**Published:** 2022-02-25

**Authors:** Yosef Tsegaye, Wasihun Admassu, Abebe Edao, Samuel Kinde, Meaza Gentu, Markos Negash, Tadelo Wondmagegn, Addisu Gize, Martha Zewdie, Kidist Bobosha, Liya Wassie

**Affiliations:** ^1^ Department of Medical Laboratory Science, Addis Ababa University, Addis Ababa, Ethiopia; ^2^ Mycobacterial Diseases Research Directorate, Armauer Hansen Research Institute, Addis Ababa, Ethiopia; ^3^ Immunology and Molecular Biology Unit, Jimma University Medical Center, Jimma, Ethiopia; ^4^ Department of Immunology, University of Gondar, Gondar, Ethiopia; ^5^ Department of Microbiology, St Paul’s Hospital Millennium Medical College, Addis Ababa, Ethiopia

**Keywords:** hormones, antibodies, tuberculosis, immunity, pathogenesis

## Abstract

Effective control of *Mycobacterium tuberculosis* (*Mtb*) infection is mediated by multifaceted factors that involve both the endocrine and immune system. Profiling hormones and antibodies in different stages of TB provides insight in the pathogenesis of the disease. In this study, we profiled endocrine hormones (dehydroepiandrosterone (DHEA), cortisol, testosterone, estradiol, growth hormone and leptins) and *Mtb* strain H37RV lipoarabinomannan (LAM)-specific antibody levels in plasma samples, collected from pulmonary TB (PTB) patients, TB lymphadenitis (TBLN) patients and latently infected (QFT-positive) or uninfected (QFT-negative) apparently healthy individuals using ELISA. Plasma levels of leptin and DHEA were significantly low in PTB and TBLN patients compared to healthy controls (P<0.0001 and P=0.02, respectively), whereas these levels significantly increased following anti-TB treatment (P=0.002 and P=0.0001, respectively) among TB patients. The levels of estradiol and testosterone significantly improved following anti-TB treatment (P=0.03 and P=0.0003, respectively), whereas cortisol and growth hormones declined significantly (P <0.05). Similarly, LAM-specific IgG, IgM and IgA were significantly higher in PTB patients compared to other groups, whereas levels of IgG1 subtype were significantly higher among LTBI groups compared to both TB patients and QFT-negative individuals (P<0.0001). Overall, we observed significantly variable levels of endocrine hormones as well as immunoglobulins across the spectrum of TB illness and such profiling has a significant contribution in selection of effective biomarkers that have roles in TB treatment monitoring or diagnostics. Although this study did not show a functional association between hormones and antibodies, alterations in the levels of these biomarkers suggest the key roles these markers play in TB pathogenesis.

## Introduction

The spectrum of tuberculosis (TB) pathogenesis often lies on interplay between the bacterium causing the illness, *Mycobacterium tuberculosis* (*Mtb*), and the host defense system, which is also maneuvered by cells of the immune system. In both experimental models and human patients, hormones are shown to act as immunomodulators and play a significant role in the pathogenesis of a disease (1). Hormones and the immune system share receptors and ligands that interact through cytokine- and hormone-producing cells, influencing the course of infection processes (2). Studies have demonstrated that specific hormones, such as cortisol, growth hormones and dehydroepiandrosterone (DHEA) correlate with tuberculosis (TB) treatment outcomes (3, 4). On the other hand, although antibody responses are often regarded as inferior to cell-mediated immunity, their role in protection against TB has been demonstrated in several studies (5–9). Profiling these two components of the body’s defense system across a spectrum of TB illness is important to better understand TB pathogenesis and provide an additional insight to the ongoing biomarker pool investigations in TB control efforts. Tuberculosis is often characterized by a spectrum of infection stages and illnesses (10). In this study, we aimed to profile levels of lipoarabinomannan (LAM)-specific immunoglobulins (Igs) and selected hormones in different spectra of *Mtb* infections and/or TB diseases (latently un/infected individuals and TB patients) using enzyme-linked immunosorbent assay (ELISA). Lipoarabinomannan, a glycolipid, is a significant part of mycobacterial surface antigen, having roles in virulence, pathogenesis, and known to elicit immunological response (11). Its repetitive D-arabinofuranose residues allows it to direct B cell stimulatory functions (12), making it a suitable antigen choice in our study.

## Materials and Methods

### Study Setting

A pool of 200 plasma samples, collected between 2005 and 2013, were systematically retrieved (based on quality, adequate sample availability, and linked clinical and socio-demographic data) from a biorepository at the Armauer Hansen Research Institute (AHRI) laboratory. Of these, 40 newly diagnosed microscopy or culture confirmed pulmonary TB (PTB) patients (before and after anti-TB treatment), 40 TB lymphadenitis patients (TBLN), 40 latently infected (QuantiFERON (QFT) TB Gold test -positive) and 40 uninfected (QFT-negative) individuals were randomly retrieved. Based on the repository database, all samples were collected from a cohort of voluntary, adult, HIV negative individuals, who resided in Addis Ababa, the capital city of Ethiopia, Hossana and Butajira areas, (which are ~230 Km and 130 Km, respectively, southwest of Addis Ababa). Majority of the PTB patients had cough (38/40), night sweating (32) and hemoptysis in a few cases (8/40). Similarly, night sweating was the most common symptom exhibited (22/40) in TBLN cases, while cough (5/40) and hemoptysis (4/40) were rare occurrences. The most commonly observed sites of lymph node enlargements in TBLN cases were cervical area (12/40) and neck (11/40); however, other sites including inguinal, submandibular, clavicular and axial sites were also involved. Additional datasets are summarized in **Table 1**. Samples with a clinical history of severe malnourishment, anemia and other debilitating illnesses were excluded during the initial screening and enrollment of participants. Also, samples with icteric and turbid plasma were not included in these analyses.

**Table 1:** Demographic and clinical features of study participants.

Parameter		Study groups			
		QFT-negative (n = 40)	QFT-positive (n = 40)	PTB (n = 40)	TBLN (n = 40)
**Age (years mean ± SD)**		20.95 ± 7.6	24.5± 9.7	26.3 ± 7.8	29.61 ± 14.5
**Sex**	**Male (N, %)**	22 (55)	22 (55)	22 (55)	12 (30)
	**Female (N, %)**	18 (45)	18 (45)	18 (45)	23 (57.5)
					5 (12.5)*
**Presence of BCG scar (N, %)**		17 (42.5)	13 (32.5)	6 (15)	5 (12.5)
**Body mass index (kg/m^2^) (mean ± SD)**		19.0 ± 3.2	20.2 ± 4.2	18.3 ± 3	Not Available

QFT, QuantiFERON; PTB, pulmonary tuberculosis; TBLN, tuberculosis lymphadenitis; *data not available.

### Plasma Sample Preparation and Quantification of Hormone and Antibody Concentrations Using Enzyme-Linked Immunosorbent Assay (ELISA)

Once retrieved from -80°C freezers, plasma samples were thawed on ice and spun at 1500 rpm for 5 minutes to transfer clear plasma supernatants into new collection tubes that were kept at 4°C until assayed by enzyme-linked immunosorbent assay (ELISA). Concentrations of hormones (DHEA, cortisol, testosterone, estradiol, growth hormone and leptin) or LAM-specific antibody (IgG, IgM, IgA or IgG subclasses) levels were measured using ELISA according to the manufacturer’s instructions (Eagle Bioscience, Italy).

Briefly, 96-well ELISA plates, pre-coated with hormones of interest (Eagle Bioscience, Italy), were incubated with plasma samples either at room temperature or at 37°C, depending on the hormones of interest and washed with diluted phosphate buffer saline (PBS) containing 0.05% Tween 20, followed by addition of enzyme-substrate conjugates for antibody binding. Similarly, Immulon I 96-well high-binding polystyrene microtiter plates (Thermo Fisher Scientific, U.S.A.) were coated with 1μg/ml of *M. tuberculosis* strain H37RV purified mannosylated lipoarabinomannan (LAM) (generously donated by BEI Resources, https://www.beiresources.org/) in 0.05M carbonate/bicarbonate coating buffer (pH 9.6) and incubated overnight at 4°C. The following day, plates were washed with PBS containing 0.1% of Tween-20 and blocked using 0.1% bovine serum albumin (BSA). Then plasma samples and standards were added into wells after dilution (for Ig analyses sample dilutions ranged from 1:25,000 to 1:100), followed by addition of enzyme-substrate conjugates for antibody binding. In both cases, a color change was developed that was later captured as optical density (OD) using an ELISA reader (reading at 450 ηm against a reference wavelength at 620 ηm). All laboratory assays were done using commercially available kits following the manufacturers’ instructions and standard operating procedures. Results were also interpreted according to the manufacturers’ instructions. Plasma concentrations of hormones or antibodies were finally extrapolated from the standard curve and all statistical analyses were done using extrapolated values from the standard curves, assuming best curve fit (with highest R values). For diluted samples, final concentrations were calculated by multiplying the extrapolated concentration with dilution factor.

### Ethics Approval

This study was performed in accordance with the Declaration of Helsinki and approved by the AHRI/ALERT Ethics Review Committee (Ref. No. PO07/18 and PO08/18), by the ethics committees of the Addis Ababa University, Department of Medical Laboratory Sciences (Ref. No. DRERC/326/18/MLS) and School of Biomedical and Laboratory Science, University of Gondar (UOG). Only anonymized samples were used to ensure privacy and confidentiality of study participants. In addition, institutional permission was obtained from AHRI to access biorepository samples and linked archived data.

### Statistical Analyses

In this study, all plasma levels of hormones (cortisol, DHEA, growth hormone, estradiol, testosterone and leptin) and Igs (IgG, IgG1, IgG2, IgG3, IgA, IgM) are presented in concentrations and were considered as dependent variables, whereas variables including clinical presentations and socio-demographic data (such as age, sex, BMI, BCG vaccination status) were considered as independent variables. Statistical analyses were done using GraphPad Prism 7.01 and SPSS version 26: comparisons between groups were done using non-parametric statistics (One-way ANOVA (Kruskal-Wallis test) followed by Dunnett’s multiple test comparison). Correlation between hormones and Ig levels with clinical status were done using Spearman correlational analysis. To compare hormone profiles or Igs in PTB patients before and after treatments, a nonparametric, Wilcoxon matched-pairs signed rank test was used. Multivariate analysis was also used to compare Ig levels between groups. Data are summarized using descriptive statistics, frequency tables and figures and a P-value less than 0.05 was considered statistically significant.

## Results

### Plasma Levels of Hormones Among the Study Groups

**Figure 1:**
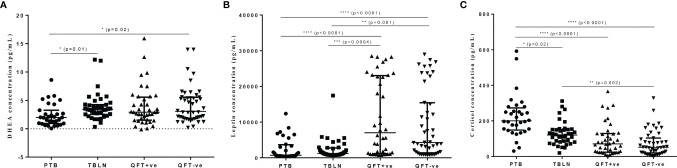
Plasma levels of selected hormones (DHEA, Leptin and Cortisol) in different groups of participants as measured by ELISA. Error bars show Median with Interquartile Range (IQR). PTB, pulmonary tuberculosis patients; TBLN, tuberculosis lymphadenitis patients; QFT-positive, QuantiFERON positive (latently infected individuals); QFT-negative, QuantiFERON negative (*Mtb* uninfected individuals). *P < 0.05, **P = 0.002, ***P = 0.0004, ****P < 0.0001.

The levels of DHEA were significantly lower in PTB patients compared to QFT-negative and TBLN patients (P<0.02 and P=0.01, respectively) (**Figure 1A**). Leptin level was also significantly lower in PTB and TBLN patients compared to apparently healthy individuals who were either QFT-positive or negative (**Figure 1B**). On the other hand, plasma cortisol level was significantly higher in PTB and TBLN patients compared to apparently healthy groups, while its level was even higher in PTB patients compared to the TBLN cases (**Figure 1C**). In a similar way, age and sex adjusted plasma levels of testosterone, estradiol and growth hormone levels were measured; however, no apparent difference was observed between the different study groups (Data not shown).

### *M. tuberculosis* LAM-Specific Antibody Profiles Among the Study Groups

**Figure 2:**
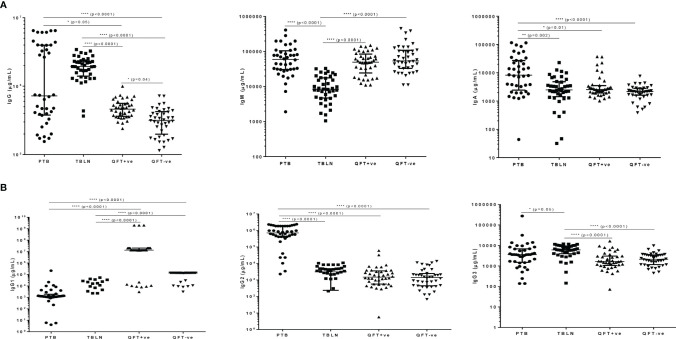
*M. tuberculosis* LAM-specific antibody responses in different groups of participants as measured by ELISA; **(A)** shows levels of IgG, IgM and IgA and **(B)** shows levels of IgG subtypes (IgG1, IgG2 and IgG3) error bars indicate Median with Interquartile Range (IQR); for better visualization of the dot plots, all graphs are shown in log scale; PTB, pulmonary tuberculosis patients; TBLN, tuberculosis lymphadenitis patients; QFT-positive, QuantiFERON positive (latently infected individuals); QFT-negative, QuantiFERON negative (*Mtb* uninfected individuals). *P < 0.05; **P = 0.002; ****P < 0.0001.

In a similar trend, we compared levels of concentrations of immunoglobulins in plasma of TB patients (both PTB and TBLN) and apparently healthy individuals, who were either latently infected (QFT-positive) or non-infected (QFT-negative). We observed a significant difference in the levels of IgG, IgM and IgA (**Figure 2A**), where PTB patients exhibited higher levels of the immunoglobulins compared to the other study groups. Similarly, levels of IgG subtypes, particularly IgG2 and IgG3, were relatively higher in PTB patients (**Figure 2B**). On the other hand, all the apparently healthy groups (regardless of the QFT responses) had higher levels of IgG1 subclass compared to both TB patient groups (P<0.0001) (**Figure 2B**).

Multiple regression was performed between hormone levels and clinical and other demographic variables (presence or absence of BCG scar, age, sex, BMI); however, no apparent association was observed, except for cortisol, whose levels poorly correlated with age (R^2 =^ 0.24, p=0.004), sex (R^2 =^ 0.24, p=0.04) and BMI (R^2 =^ 0.24, p=0.0001) and testosterone, whose levels strongly correlated with BMI (R^2 =^ 0.670, P=0.0001). A similar regression analysis between antibody levels and socio-demographic characteristics showed no apparent associations between the two variables; however the associations were more visible between antibody levels and types with underlying mycobacterial infections and disease states as shown in **Figure 2**. On the other hand, a Spearman correlation analysis was performed between levels of LAM-specific Ig responses and presence or absence of BCG scar and the analyses indicated variable degrees of correlation between Ig levels (particularly IgG, IgM, IgA and IgG2) and presence of BCG scar with correlation coefficients (rho=0.65, P<0.0001; rho=0.59, P<0.0001; rho=0.39, P=0.005 and rho=0.59, P< 0.0001, respectively). However, a similar analysis between hormone levels and some of the independent variables showed no correlation between hormones levels and other variables (Data not shown).

### Impact of Anti-TB Treatment on Plasma Levels of Hormones and LAM-Specific Immunoglobulins in PTB Patients

**Figure 3:**
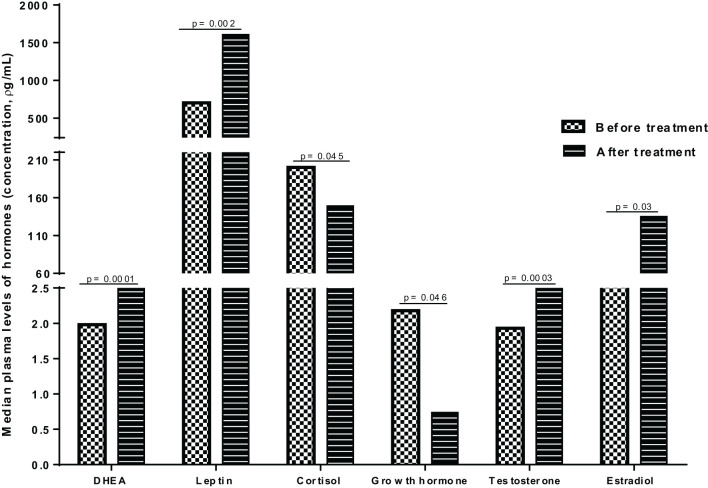
Median plasma levels of hormones in PTB patients before and after anti-TB treatment.

To assess the impact of anti-TB treatment on plasma hormone concentrations, we compared the median plasma levels of hormones in PTB patients at baseline (before initiation of anti-TB treatment) and following completion of treatment (after 6 months of therapy) and observed significant alterations in the plasma levels of hormones after anti-TB treatment (**Figure 3**). The median levels of DHEA, leptin, testosterone and estradiol were significantly elevated following completion of treatment, whereas there was an apparent decline in the median levels of cortisol and growth hormones after treatment completion (**Figure 3**).

Similarly, plasma antibody levels were compared in PTB patients before and after completion of anti-TB treatment; however, no apparent difference was observed, except for IgM, where the levels significantly declined following anti-TB treatment (P=0.001) (Data not shown).

## Discussion

How hormones interact with the immune systems dictate the way optimal immune responses are also induced during host-pathogen interactions (1). Such optimal responses are often triggered through changes in the secretion patterns of hormones as well as the immune system. The main aim of this study was to profile hormone and antibody responses and measure how their levels alter during a spectrum of illnesses such as TB (latent TB infection, pulmonary and extapulmonary TB disease or following treatment completion). Though cell-mediated immunity demonstrates a major role in protection against TB, antibodies are also recently reported to play a significant role in bacterial clearance and enhanced phagolysosomal maturation during TB infection (7). In this study, we profiled plasma levels of selected endocrine hormones (cortisol, dehydroepiandrosterone, leptin, human growth hormone, testosterone, and estradiol) and antibodies (IgG, IgG1, IgG2, IgG3, IgA, IgM) in tuberculosis patients with varied forms of the disease (pulmonary TB and TB lymphadenitis) and in comparison to apparently healthy individuals, who were further categorized as latently infected or uninfected.

The findings of our study suggest that active TB disease is characterized by elevated plasma levels of cortisol and decreased DHEA, resulting in a greater cortisol/DHEA ratio when compared to both the healthy controls and latently infected groups. This has been also evident from earlier studies, where cortisol level was significantly higher in TB patients compared to healthy individuals and that this increased level also correlated with disease severity, whereas it was the reverse for DHEA (13, 14). Previous studies also showed that cortisol promotes T-helper 2 (Th2) immune activity, partly by suppressing Th1 cells and that its natural antagonist, DHEA, is shown to promote Th1 cytokine production, while interfering with Th2 cytokine synthesis (15, 16). Because infection with intracellular pathogens such as TB necessitates the expression of Th1 cytokines such as IFN-γ, a central macrophage-activating cytokine that is also involved in immune protection against *MTB* (17), an increase in plasma levels of cortisol and a decrease in DHEA levels may impair the immune response against TB, favoring increased susceptibility and disease progression (18).

Furthermore, the plasma levels of cortisol and DHEA were considerably altered following therapy, with cortisol levels dramatically decreasing and DHEA increasing. Others have also reported a similar finding that cortisol levels decline throughout TB treatment in cured patients but remain unchanged in patients with a failed treatment outcome, whereas DHEA levels steadily increase in the cured groups during treatment (3). These changes demonstrate the importance of these hormones in the immunopathogenesis of tuberculosis. Similar alterations have been also reported from animal studies, where levels of DHEA improved, following treatment with DHEA sulfate, boosting the production of IFN-γ (19).

Similar like other reports (14, 20, 21), the TB patients had considerably lower plasma levels of leptin when compared to both latently infected and uninfected groups. However, this level significantly improved in PTB patients following anti-TB treatments (3). On the other hand, a few other studies have reported an increase or no change in leptin levels in TB patients (22, 23). The reasons for this disparity could be related to nutritional or dietary factors, genetic, environmental or treatment initiation time. Other studies have also demonstrated the inhibitory role persistent activation of pro-inflammatory cytokines play on leptin synthesis in TB patients (24). Because leptin concentration is also linked to adipose tissue mass (25), the lower leptin levels seen in TB patients and its change after therapy could be linked to disease-associated weight loss and the association between leptin levels and wasting seen in most TB patients.

Our analysis on the plasma levels of sex hormones (testosterone and estradiol) showed no apparent difference among the study groups; however, others have shown a considerably lower testosterone levels in TB patients (4, 13), which was also associated with other pro-inflammatory cytokines such as IFN-y, IL-6, and TGF-β (26). However, there was a significant alteration in the levels of both testosterone and estradiol concentrations in PTB patients following anti-TB treatment and this was also consistent with earlier reports (3). Although no significant difference was observed in the levels of growth hormones (GH) between the study groups, the level significantly declined after treatment in PTB patients. However, an earlier study showed higher levels of GH in TB patients compared to healthy individuals (14). On the other hand, others reported the roles GHs play in stimulating human monocytes to destroy *Mtb* by serving as a human macrophage-activating factor (27).

Similarly, plasma immunoglobulin levels were measured in individuals exhibiting different clinical presentations and exposure to TB and here *Mtb-derived* LAM was used as an immunogen to examine specific Ig levels. The findings of our study indicate that there is a significant difference in antibody profiles in different disease states, possibly suggesting for their role as potential biomarkers in future vaccines or diagnostics developments (28, 29). While there are no tools to specifically distinguish latent TB infection from active disease, some of these markers, particularly LAM-specific IgG and IgA responses, seem to play that role in TB, as also reported by others (5, 29).

This difference in the levels of IgG might not be the same, however, if HIV becomes a factor, as reported earlier (30), where HIV-positive TB patients exhibited a significantly lower LAM-specific IgG subclasses compared to non-infected TB patients. Similarly, PTB patients exhibited a significantly higher levels of LAM-specific IgA compared to apparently healthy controls, who were either QFT-positive or negative. Earlier studies have also reported significantly higher levels of *Mtb* antigen-specific IgA responses in TB patients compared to healthy controls (31–34). In the current study, latently infected individuals showed a higher level of IgG1 titer, whereas earlier studies reported higher IgG1 and IgG2 levels in PTB patients (35), indicating their functional role during TB disease (9). The increased levels of IgG1 subtype in LTBI groups indicate its distinct glycosylation characteristics that influence binding of Fc receptors on effector cells, which may play roles during bacterial containment (40).

In this study, we also compared the breadth of the antibodies in PTB patients before and after treatment and the levels of LAM-specific IgM significantly dropped following treatment, possibly implying for the impact of treatment on *Mtb* clearance and containment of active infection. This also possibly implies to the role of IgM as a useful biomarker for treatment monitoring in TB patients.

In conclusion, lower levels of anabolic hormones such as DHEA and leptin and elevated levels of cortisol could mediate the gradual debilitation and other disabling symptoms associated with TB. Furthermore, the observation that anti-LAM Ig responses (as shown in antibody titer, type and subtype) differ significantly during TB stages, indicates a valuable roles these antibodies play during TB pathogenesis. The altered plasma levels of hormones as well as immunoglobulins during treatment suggest that evaluating these biomarkers could have prognostic importance, particularly in the evaluation of treatment outcomes, biomarker research and bacterial containment. Overall, profiling such biomarkers in a spectrum of an illness, particularly in a disease like TB, will have a significant contribution in future biomarker evaluations for treatment monitoring or diagnostics. However, the observed differences in the levels of these markers could not account for intrinsic factors related to the use of repository specimens, missing data on dietary intake or menstrual cycles/ovulation, particularly in female participants. This study does not show the functional association between hormones and antibodies; however, the observed changes in the levels of these biomarkers could possibility indicate their key roles in TB pathogenesis. A large-scale study might therefore be warranted to better understand the interaction between the endocrine and the immune system, particularly the innate immunity.

## Data Availability Statement

The raw data supporting the conclusions of this article will be made available by the authors, without undue reservation.

## Ethics Statement

The studies involving human participants were reviewed and approved by The AHRI/ALERT Ethics Review Committee, The ethics committee of the Addis Ababa University, Department of Medical Laboratory Sciences and School of Biomedical and Laboratory Science, University of Gondar. The patients/participants provided their written informed consent to participate in this study.

## Author Contributions

YT and WA did all the laboratory work, analyzed data, and wrote the 1^st^ draft of the manuscript. AE, SK, MG, MN, TW, AG, MZ, and KB analyzed the data, edited the manuscript, and supervised the work. LW conceived and designed the study, analyzed the data, supervised the study, and edited the manuscript. All authors contributed to the article and approved the submitted version.

## Funding

This work was supported by EDCTP grant through Collaboration and Integration of Tuberculosis Vaccine Trials in Europe and Africa (TBTEA) Consortium [grant number MS. 2010.18000.002] and core funds from Armauer Hansen Research Institute (AHRI), received from SIDA and NORAD. The funders had no role in the study design, data collection and analysis, decision to publish, or preparation of the manuscript.

## Conflict of Interest

The authors declare that the research was conducted in the absence of any commercial or financial relationships that could be construed as a potential conflict of interest.

## Publisher’s Note

All claims expressed in this article are solely those of the authors and do not necessarily represent those of their affiliated organizations, or those of the publisher, the editors and the reviewers. Any product that may be evaluated in this article, or claim that may be made by its manufacturer, is not guaranteed or endorsed by the publisher.
